# Streamlining efficient and selective synthesis of benzoxanthenones and xanthenes with dual catalysts on a single support

**DOI:** 10.1038/s41598-023-43746-y

**Published:** 2023-09-30

**Authors:** Najmedin Azizi, Fezzeh Farzaneh, Elham Farhadi

**Affiliations:** Chemistry and Chemical Engineering Research Center of Iran, P.O. Box 14335-186, Tehran, Iran

**Keywords:** Catalysis, Green chemistry, Organic chemistry

## Abstract

Using two catalysts on a single support can improve reaction efficiency, higher yields, improved selectivity, and simplified reaction conditions, making it a valuable approach for industrial transformation. Herein, we describe the development of a novel and effective heterogeneous catalyst, WCl_6_/CuCl_2,_ supported on graphitic carbon nitride (W/Cu@g-C_3_N_4_), which was synthesized under hydrothermal conditions. The structure and morphology properties of the W/Cu@g-C_3_N_4_ were characterized using various spectroscopic techniques, including FTIR, XRD, TEM, TGA, EDX, and SEM. The W/Cu@g-C_3_N_4_ support material enabled the rapid and efficient synthesis of benzoxanthenones and xanthenes derivatives in high yields under mild reaction conditions and short reaction times. The W/Cu@g-C_3_N_4_ catalyst was also found to be easily recyclable, and its catalytic performance did not significantly decrease after five times use. The findings suggest that W/Cu@g-C_3_N_4_ is a promising chemical synthesis catalyst with significant implications for sustainable and cost-effective organic synthesis.

## Introduction

Recently, xanthenes and their derivatives have attracted considerable attention due to their potential applications in various fields, including biological and pharmaceutical sciences and laser technology^1,2^. These compounds have been found to exhibit a range of beneficial properties, such as anti-inflammatory, antimicrobial, antiviral, and antitumor activities, making them attractive targets for drug development^3,4^. Several methods for the preparation of biologically important 14-aryl-14H-dibenzo[a,j]xanthenes and 1,8-dioxo-octahydroxanthenes have been reported in the literature^5–8^, including the use of various catalysts or promoters^9–11^. However, most acid catalysts are highly corrosive and difficult to recover for subsequent reactions, which can limit their practical application^12^. To address this issue, there is a growing interest in developing green and reusable catalysts using simple and inexpensive starting materials for the environmentally safe synthesis of benzoxanthenone derivatives^13–17^. However, using acid catalysts in synthesizing benzoxanthenone products has several drawbacks, such as being highly corrosive and difficult to recover for subsequent reactions. Therefore, developing green and reusable catalysts has become increasingly crucial for the environmentally safe synthesis of benzoxanthenone derivatives. These catalysts should be made from simple, inexpensive starting materials and offer excellent catalytic activity, selectivity, and reusability. Developing such catalysts can provide a sustainable and cost-effective approach to chemical synthesis while minimizing the environmental impact.

Graphite carbon nitride (g-C_3_N_4_) is a polymeric material composed of carbon and nitrogen with a graphite-like structure^18^. It can be easily produced from inexpensive materials such as cyanamide, dicyanamide, melamine, urea, and thiourea via thermal condensation at around 550 °C^19^. In recent years, g-C_3_N_4_ has drawn attention for its widespread applications in catalysis, sensors, adsorption, drug delivery, gas separation, and solar cells^20–23^. As a strong support material, g-C_3_N_4_ has many acidic and basic Lewis sites, such as terminal and bridge NH– groups and N lone pairs in triazine/heptazine rings, potential sites for metal adsorption^24–26^. Additionally, g-C_3_N_4_ is highly stable against thermal and chemical attacks due to its tri-s-triazine ring structure and a high degree of condensation^27–29^. These structures resist high temperatures (up to 600 °C) and do not change their system^30–35^. Thus, g-C_3_N_4_ is a potential support material for various industrial applications^36–39^. Researchers have tried to design and fabricate nanostructures to enhance the photocatalytic activity of g-C_3_N_4_ by adjusting the size and surface properties. Furthermore, doping g-C_3_N_4_ with metal significantly improves its catalytic activity^40–42^.

## Experimental

### Materials and chemicals

All the chemicals utilized in this study were procured from commercially available sources and used without requiring additional modifications. Solvents were distilled before use. Fourier transform infrared (FT-IR) spectra were obtained using a Bruker Vector-22 infrared spectrometer with KBr pellets. Melting points were measured using a Buchi 535 melting point apparatus. The SEM and EDX analyses were performed using a TESCAN Vega Model scanning electron microscope. XRD patterns were obtained using a X'pert Pro model from Panalytical (Holland). TGA experiments were conducted using a TGA 209F1 thermoanalyzer instrument from Netzsch, (Germany). TEM of the samples were determined using a Zeiss EM10C Transmission Electron Microscope (Germany). NMR spectra were recorded Bruker Avance III HD spectrometer on a 500 or 300 MHz spectrometer for the ^1^H nucleus, and a 125.7 or 75 MHz spectrometer for the ^13^C nucleus, using CDCl_3_ or DMSO-d_6_ as solvent.

### Composite preparation

#### Preparation of g-C_3_N_4_

The g-C_3_N_4_ nanoparticles were prepared using a previously reported method^19^. First, 10 g of melamine was placed in a 40 cm × 120 cm ceramic container without a lid and heated for 3 h at 550 °C with an increased rate of 5 °C/min. After the process was completed and the temperature reached an ambient level, a yellow powder was obtained. Heating the bulk sample at 550 °C for 2 h resulted in the formation of g-C_3_N_4_ nanosheets.

#### Preparation of W/Cu@g-C_3_N_4_

1 g of g-C_3_N_4_ was dispersed in 100 mL of methanol under stirring, and then the reaction mixture was subjected to ultrasound for 10 min at ambient temperature. In the next step, 200 mg of WCl_6_ and 200 mg of CuCl_2_ were dissolved in 10 mL of methanol in a flask using a stirrer. Then, the two solutions were mixed under stirring for 10 min. After that, the reaction mixture was heated at 60 °C for 2 h. The W/Cu@g-C_3_N_4_ catalyst was obtained after completion of the reaction, then filtered and washed with methanol and dried under vacuum at 60 °C.

### General Procedure

Aldehyde (1 mmol) and either 2-naphthol or dimedone (2 mmol) were combined with W/Cu@g-C_3_N_4_ (40 mg) and stirred at 80 °C for 1–3 h without the use of a solvent. Thin-layer chromatography (TLC) was used to track the advancement of the reaction. Once the reaction was complete, ethyl acetate (30 mL) was added to the mixture, and the catalyst was separated from the reaction mixture by centrifugation. The solid product underwent recrystallization using either ethanol or ethyl acetate to achieve purification. Subsequently, all compounds were known, and their melting points were determined by comparing them with reference standards.

## Results and discussion

Recently, there has been a growing emphasis on designing more environmentally friendly and sustainable organic reactions using cheap and non-toxic catalysts^43–45^. As part of our ongoing commitment to green chemistry, we have been exploring environmentally friendly reaction media, such as water and deep eutectic solvents, to prepare natural and biocompatible materials. This study presents an efficient, green, and rapid method for synthesizing benzoxanthenones and xanthenes derivatives using W/Cu@g-C_3_N_4_ as an eco-friendly and effective catalyst in solvent-free conditions. The W/Cu@g-C_3_N_4_ nanocomposite was prepared by supporting WCl_6_/CuCl_2_ on graphitic carbon nitride under hydrothermal conditions. The graphitic structure of carbon nitride served as a direct and rapid catalyst with potential support for double metal salts.

**Figure 1:**
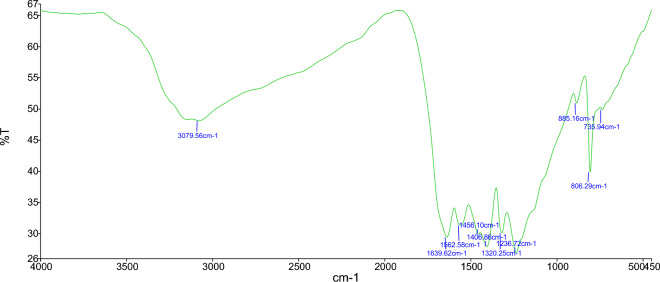
FT-IR spectrum of W/Cu@g-C_3_N_4_.

The FT-IR spectrum in Fig. 1 shows the presence of high-density triazine/heptazine rings in WCl_6_/CuCl_2_@g-C_3_N_4_. The absorption peak at 3000–3400 cm^−1^ is due to the –NH_2_ groups in the g-C_3_N_4_ or the NH stretching vibration and may also be attributed to water absorption from the air. The absorption peaks in the 1236–1639 cm^−1^ range correspond to the stretching vibrations of the carbon–nitrogen bonding groups that are efficiently incorporated in the g-C_3_N_4_ sample. Additionally, the strong absorption peak at 806 cm^−1^ indicates the bending vibration of the s-triazine rings. The FT-IR spectrum did not show any change in the prepared nanocomposite after WCl_6_ and CuCl_2_ doping on g-C_3_N_4_, indicating that the W/Cu@g-C_3_N_4_ remained stable during the synthesis of the nanoparticles.

**Figure 2:**
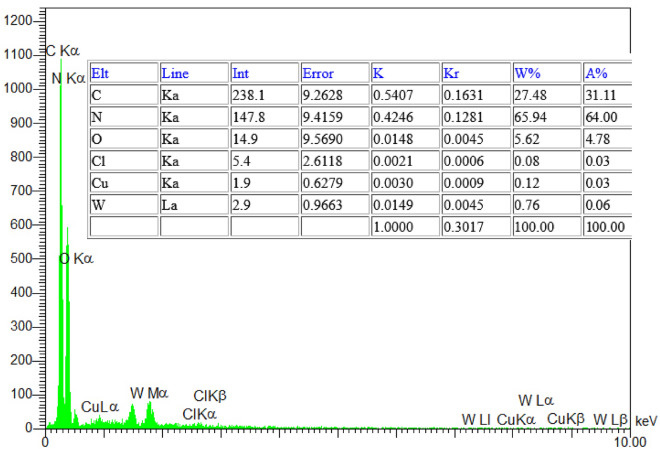
EDS spectrum of W/Cu@g-C_3_N_4_.

Energy dispersive spectroscopic (EDS) analysis, as shown in Fig. 2, was used to determine the percentage and chemical composition of WCl_6_/CuCl_2_@g-C_3_N_4_. The EDS mapping confirms the presence of C, N, W, Cu, and Cl elements in the composite. The adoption of W and Cu onto the g-C_3_N_4_ the surface was successful.

**Figure 3:**
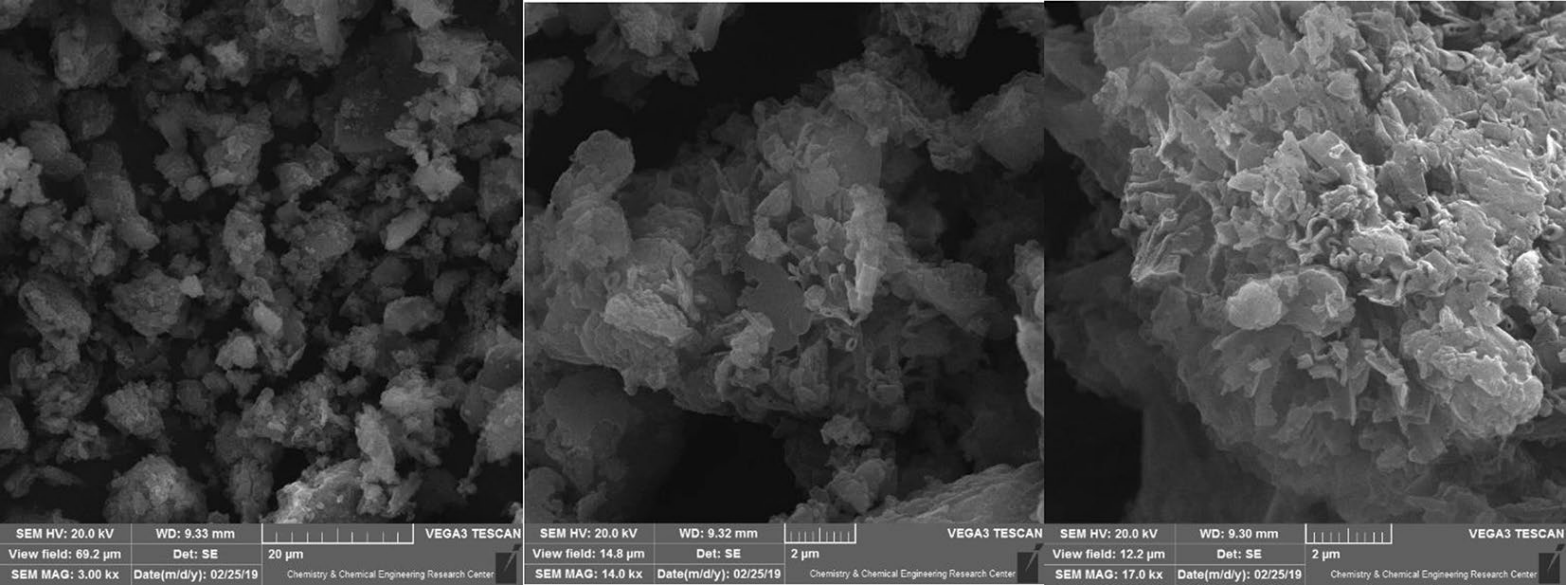
SEM images of W/Cu@g-C_3_N_4_.

To examine the surface morphology of the newly developed nanocomposite, SEM microscopy analysis was performed, and the results are presented in Fig. 3. The SEM spectrum of the W/Cu@g-C_3_N_4_ nanocomposite demonstrates that the small sheet-like layered structure has been maintained in the stacked state.

**Figure 4:**
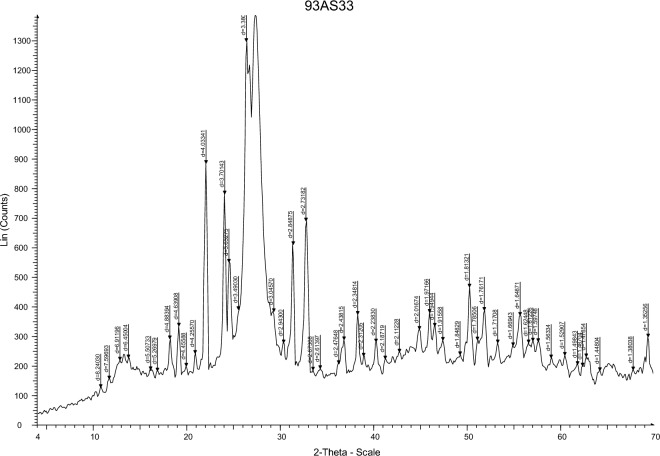
XRD patterns of the W/Cu@g-C_3_N_4_.

The W/Cu@g-C_3_N_4_ XRD characterization is shown in Fig. 4. The characteristic bending peaks at 22.3° and 27.4° belonged to W/Cu@g-C_3_N_4 ._According to the XRD analysis. The nano catalyst’s crystalline structure has been preserved despite the reaction with the WCl_6_ and CuCl_2_.

**Figure 5:**
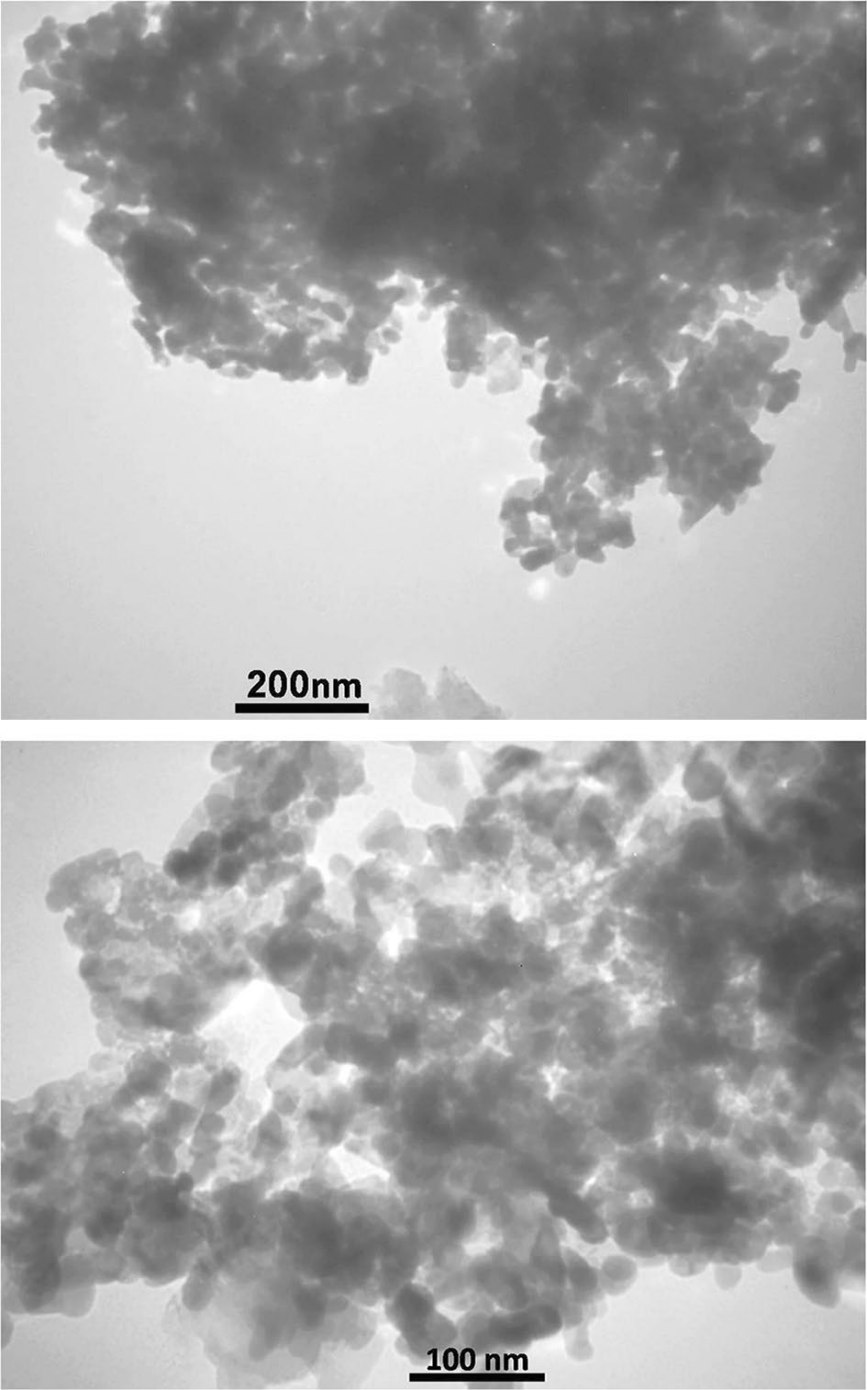
The TEM image of W/Cu@g-C_3_N_4_.

Figure 5 of the study displays TEM (Transmission Electron Microscopy) images of the W/Cu@g-C_3_N_4_ nanocomposite. In the TEM images, the g-C_3_N_4_ component is observed to have a bulk morphology with a lamellar structure at the edges. It is described as having large sheets consisting of irregular micro-fragments. The g-C_3_N_4_ appears as a grey color in the images. On the other hand, the W and Cu components are represented by irregular spheres with some agglomeration. These particles can be visually distinguished in the images due to their dark color, which contrasts with the grey color of the g-C_3_N_4_. The TEM images provide evidence that the W and Cu particles are immobilized or supported on the surface of the g-C_3_N_4_ material. This observation aligns with the findings from the SEM images.

ICP-AES (Inductively Coupled Plasma-Atomic Emission Spectroscopy) is a spectroscopic technique commonly used for the analysis of metal analytes and their concentrations in various samples, including nanocomposites. The presence and concentration of copper (Cu) and tungsten (W) in a nanocomposite were determined using a combination of EDX (Energy-Dispersive X-ray Spectroscopy) analysis and ICP-OES (Inductively Coupled Plasma-Optical Emission Spectroscopy) analysis. The EDX analysis indicated a Cu(II) content of 0.12% on the surface of the nanocomposite. To confirm the Cu(II) content obtained from the EDX analysis, ICP-OES analysis was performed. The ICP-OES test revealed a Cu(II) content of 0.10% in the nanocomposite. The close agreement between the values obtained from EDX (0.12%) and ICP-OES (0.10%) confirms the presence of Cu(II) in the nanocomposite at the given concentration. Similarly, the EDX analysis of the composite indicated a tungsten (W) content of 0.76 wt% on the nanocomposite surface. The value obtained from the ICP-OES analysis for the W loading was 0.74 wt%, which is in good agreement with the result obtained from the EDX analysis.

**Figure 6:**
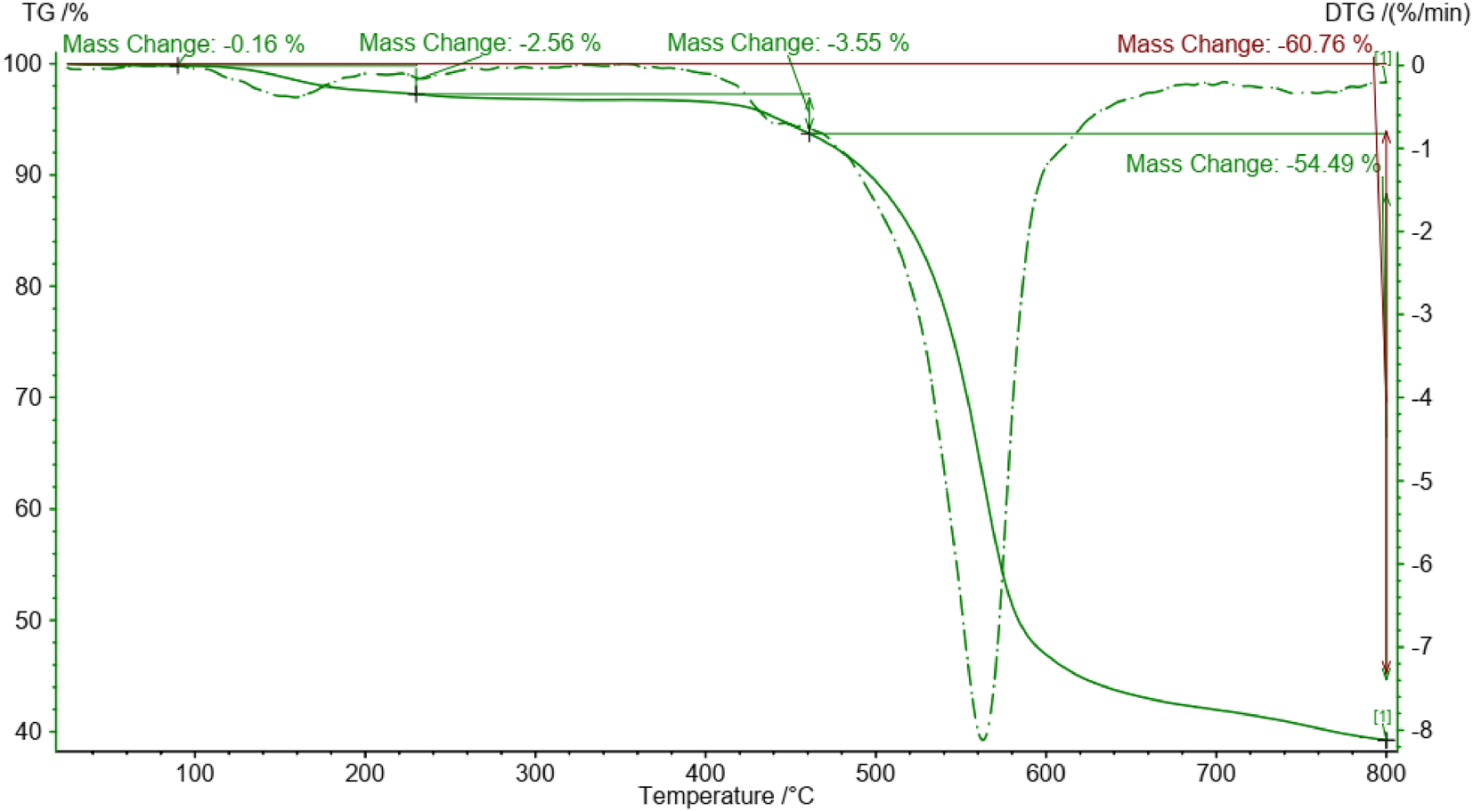
TGA diagram of the W/Cu@g-C_3_N_4_.

A TGA curve was employed to evaluate the thermal stability of W/Cu@g-C_3_N_4_, and the corresponding results are illustrated in Fig. 6. The TGA diagram indicates that the slight decrease in mass below 110 °C is related to the evaporation of water in the structure of W/Cu@g-C_3_N_4_, while the primary weight loss observed at temperatures above 400 °C is due to the decomposition of the structure. The subsequent mass loss observed above 600 °C is attributed to the decomposition of graphitic carbon nitride.

The synthesis of xanthenes and benzoxanthenones derivatives was achieved by reacting aldehyde (1 mmol) with dimedone and/or 2-naphthol (2 mmol) in the presence of W/Cu@g-C_3_N_4_ (40 mg) as the catalyst. The catalytic efficiency of W/Cu@g-C_3_N_4_ was evaluated through the reaction, and the optimal conditions are summarized in Table 1. Different parameters, such as solvent, temperature, and catalyst amount, were varied to optimize the reaction conditions. Ultimately, the highest efficiency in the shortest time was achieved with 40 mg of W/Cu@g-C_3_N_4_ at 80 °C (Table 1, entry 2). Various quantities of W/Cu@g-C_3_N_4_ were examined, and it was observed that an increased amount of catalyst (50 mg) did not result in a higher yield (Table 1, entry 7), while lower amounts (10 mg) resulted in decreased yield (Table 1, entry 10). Also, without the catalyst, only aldehyde and dimedone and Micheal addition products were recovered along with minor amount of products (12%) . Notably, the reaction was found to be unsuccessful when tested in organic solvents such as ethanol and ethyl acetate, chloroform, toluene, methanol, and water in the presence of 40 mg W/Cu@g-C_3_N_4_ (Table 1, entries 11–16). Additionally, composite elements, including g-C_3_N_4_ (Table 1, entry 17), and WCl_6_ and CuCl_2_ (Table 1, entries 18, 19) were tested independently, which led to a reduction in yield compared to the W/Cu@g-C_3_N_4_.

**Table 1:** Optimization of the synthesis of xanthenes derivatives.


Entry	Catalyst	Solvent (5 mL)	Temp. (°C)	Yields (%)^a^
1	–	Neat	80	12
2	W/Cu@g-C_3_N_4_ (40 mg)	Neat	80	92
3	W/Cu@g-C_3_N_4_ (40 mg)	Neat	100	92
4	W/Cu@g-C_3_N_4_ (40 mg)	Neat	60	64
5	W/Cu@g-C_3_N_4_ (40 mg)	Neat	40	32
6	W/Cu@g-C_3_N_4_ (40 mg)	Neat	rt	26
7	W/Cu@g-C_3_N_4_ (50 mg)	Neat	80	92
8	W/Cu@g-C_3_N_4_ (30 mg)	Neat	80	81
9	W/Cu@g-C_3_N_4_ (20 mg)	Neat	80	67
10	W/Cu@g-C_3_N_4_ (10 mg)	Neat	80	58
11	W/Cu@g-C_3_N_4_ (40 mg)	Ethyl acetate	80	51
12	W/Cu@g-C_3_N_4_ (40 mg)	Ethanol	80	31
13	W/Cu@g-C_3_N_4_ (40 mg)	Chloroform	80	48
14	W/Cu@g-C_3_N_4_ (40 mg)	Toluene	80	54
15	W/Cu@g-C_3_N_4_ (40 mg)	Water	80	23
16	W/Cu@g-C_3_N_4_ (40 mg)	Methanol	80	28
17	g-C_3_N_4_ (50 mg)	Neat	80	14
18	WCl_6_ (40 mg)	Neat	80	54
19	CuCl_2_(40 mg)	Neat	80	38
20	W@g-C_3_N_4_	Neat	80	76
21	Cu@g-C_3_N_4_	Neat	80	48

^a^Isolated yields. Reaction time: 60 min. Reaction condition: Aldehyde (1 mmol) and dimedone (2 mmol) were combined with the listed catalyst and stirred at 80 °C for 60 min.

The synthesis of xanthenes and benzoxanthenones derivatives was carried out by reacting aldehyde (1 mmol) with dimedone (2 mmol) in the presence of W/Cu@g-C_3_N_4_ (40 mg) as the catalyst. Optimization of the reaction conditions was achieved by altering various parameters, including solvent, temperature, and the amount of catalyst. The optimal conditions were performed with 40 mg of W/Cu@g-C_3_N_4_ at 80 °C, which resulted in the highest efficiency in the shortest time. After obtaining the optimal conditions, we investigated the scope and limitations of the reaction by using different aromatic aldehydes with both electron-withdrawing and electron-donating groups. Upon obtaining the optimal conditions, we assessed the scope and limitations of the reaction by utilizing various aromatic aldehydes with both electron-withdrawing and electron-donating groups. By condensing various aromatic aldehydes with dimedone under the selected conditions, good to excellent yields were achieved within short reaction times, as summarized in Table 2. Aromatic aldehydes with electron-donating groups showed no significant difference in yield and reaction time compared to those with electron-withdrawing groups.

**Table 2:**
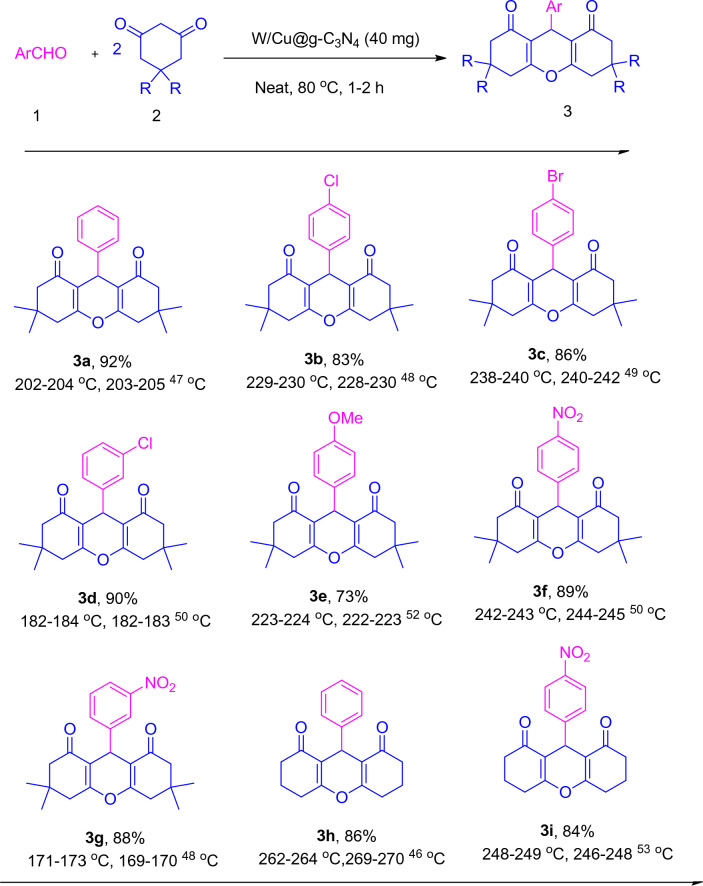
The synthesis of 1,8-dioxo-octahydroxanthenes in the presence of W/Cu@g-C_3_N_4_ as a catalyst.

^a^Isolated yields. Aldehyde (1 mmol) and either dimedone (2 mmol) were combined with W/Cu@g-C_3_N_4_ (40 mg) and stirred at 80 °C for 1–2 h.

After the successful application of W/Cu@g-C_3_N_4_ in the preparation of 1,8-dioxo-octahydroxanthenes, the catalyst's application scope in the synthesis of 14-aryl-14H-dibenzo[a,j]xanthenes was evaluated. Consistent with the prior reaction, the same reaction conditions were maintained, leading to favorable outcomes such as high yield, short reaction time, and excellent performance. The intended reaction was carried out by reacting aldehyde (1 mmol) with 2-naphthol (2 mmol) in the presence of W/Cu@g-C_3_N_4_ catalysts. 40 mg of catalyst was used for this reaction at a temperature of 110 °C, and 14-aryl-14H-dibenzo[a,j]xanthenes were obtained with a yield of 90% in 60 min.

To investigate the generality and domain of this method, different aromatic aldehydes with different structures were used in the model reaction. The results obtained from the reaction are presented in Table 2, where it can be observed that various aldehydes with both electron-rich and electron-deficient groups yielded good to excellent results (68–90%). Moreover, heteroaromatic aldehydes such as pyridine-4-carbaldehyde, pyridine-2-carbaldehyde, and thiophene-2-carbaldehyde also acted well as substrates in the reaction, leading to high efficiency in converting to dibenzo[a,j]xanthenes (Table 3).

**Table 3:**
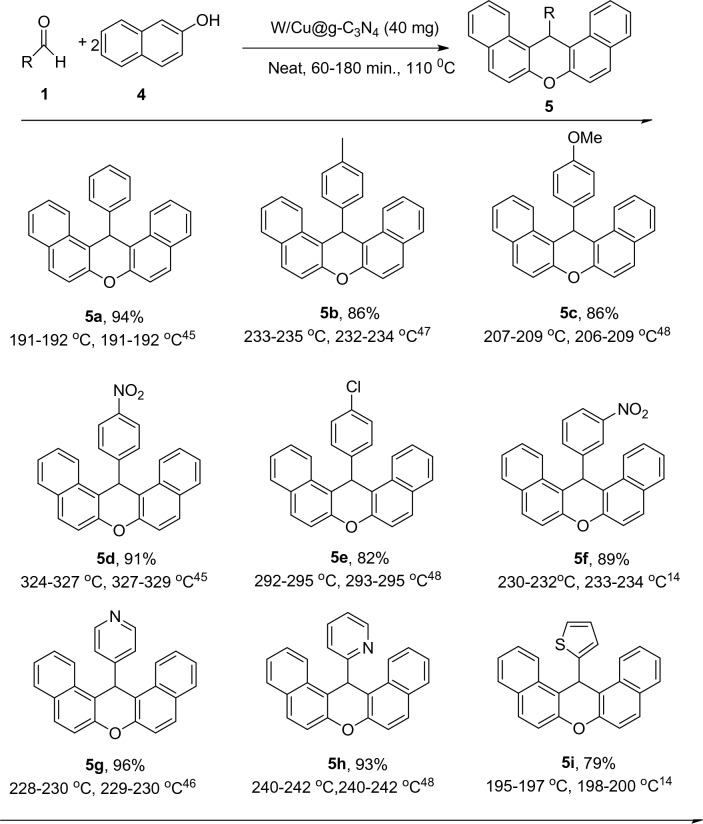
The synthesis of 14-aryl-14H-dibenzo[a,j]xanthenes in the presence of W/Cu@g-C_3_N_4_ as a catalyst.

^a^Isolated yields. Aldehyde (1 mmol) and either 2-naphthol (2 mmol) were combined with W/Cu@g-C3N4 (40 mg) and stirred at 110 °C for 1–3 h.

**Figure 7:**
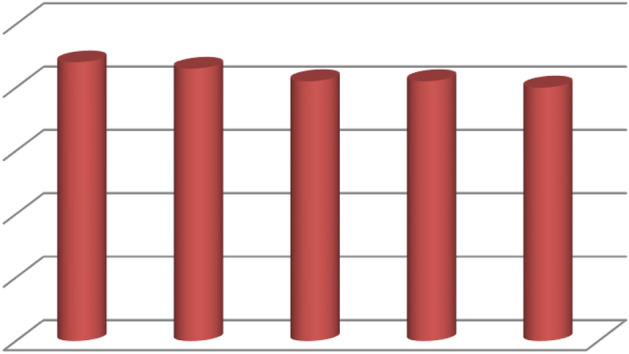
Recyclability of catalyst.

To investigate the practical application of W/Cu@g-C_3_N_4_ in the synthesis of xanthenes derivatives, the efficiency and strength of the recycled catalyst were examined in a model reaction, as presented in Table 1. In order to minimize reaction errors, the reaction was conducted on a 5 mol scale, using 200 mg of catalyst for each run. Once the reaction was completed, the reaction mixture was cooled to room temperature, and ethyl acetate (30 mL) was added. The catalyst was then separated with the assistance of a centrifuge and washed with hot ethyl acetate. Afterward, it was dried. This procedure was repeated for four consecutive runs, and the results are depicted in Fig. 7. The findings demonstrated that W/Cu@g-C_3_N_4_ could be recycled and reused at least five times without experiencing a significant reduction in yield, as depicted in Fig. 7. This indicates that the catalyst exhibits good stability and can be effectively utilized in future reactions. Consequently, it represents a practical and cost-effective option for large-scale synthesis of xanthenes derivatives.

To provide additional confirmation of the stability of the W/Cu@g-C_3_N_4_ catalyst, several analytical techniques were employed, including TGA, EDX and TEM analysis. Both the fresh and recycled catalyst samples were subjected to these analyses. The TGA analysis revealed no significant changes in the weight loss behavior of the recycled W/Cu@g-C_3_N_4_ catalyst after five cycles. The FTIR spectra of the fresh and recycled catalyst samples displayed no appreciable differences in their chemical structures. TEM analysis provided visual evidence of the catalyst's structural integrity. The images of the fresh and recycled W/Cu@g-C_3_N_4_ catalyst showed no apparent changes in morphology or particle distribution. EDX analysis was conducted to evaluate the elemental composition of both the fresh and recycled catalyst samples. The results revealed a minor change in the composition of the catalyst after five cycles. Specifically, the element chlorine (Cl) was found to be absent in the recycled catalyst, indicating its removal or depletion during the recycling process.

The catalytic activity of W/Cu@g-C_3_N_4_ was evaluated and compared to previously reported reaction conditions for the production of benzoxanthenones. The results of this comparison are presented in Table 4. Based on the data in Table 4, it can be observed that the current protocol demonstrates higher efficiency and environmental friendliness compared to the previously reported methods. The new protocol offers several advantages, including a shorter reaction time, a reusable catalyst system, and a straightforward work-up procedure^46–59^.

**Table 4:** A Table of comparison with reported methods in the literature.

Entry	Catalyst	Reaction conditions	Reusability of catalyst	Yield (%)	References
1	Sulfated polyborate	Neat (no solvent) at 100 °C; for 0.05 h	1	99	^49^
2	silica-bonded S-sulfonic acid	Ethanol, 10 h; Reflux	1	98	^50^
3	manganese dihydrogen phosphate dihydrate	Neat (no solvent) at 100 °C; 0.25 h	1	98	^51^
4	nano-zirconia-supported on sulfonic acid	Neat (no solvent) at 100 °C; 2 h	3	94	^52^
5	[bmim]HSO_4_	[bmim]HSO4 100 °C; 0.416667 h		93	^53^
6	dimethyldodecylammonium)propanesulfonic acid hydrogen	Water at 100 °C; for 1 h	1	93	^54^
7	1,3,5-trichloro-2,4,6-triazine;;	Water, 120 °C; 0.833333 h	1	92	^55^
8	envirocat EPZ-10	water 70 °C; 2.5 h;	1	92	^56^
9	grafting of sulphamic acid on functionalized sawdust	Ethanol 0.833333 h; Reflux	1	92	^57^
10	H3[PW7Mo5O40].12H2O on bentonite	Neat (no solvent) 80 °C; 0.0833333 h	1	92	^58^
11	toluene-4-sulfonic acid	Acetonitrile 80 °C; 1.16667 h	1	74	^59^
12	W/Cu@g-C3N4	Neat (no solvent) 80 °C; 1 h	5	92	This work

**Figure 8:**
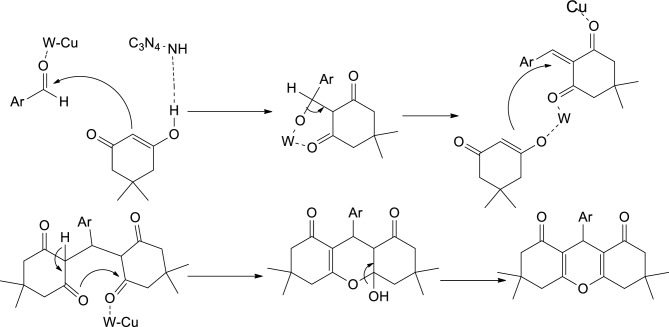
Proposed reaction mechanism.

The proposed reaction sequence in Fig. 8 provides a possible explanation for the formation of compound **3**. According to the proposed reaction sequence, the catalytic system involving Cu and W on g-C_3_N_4_ synergistically activates carbonyl groups, acting as highly active Lewis acids. Initially, a Knoevenagel condensation reaction occurs between the activated aldehydes and dimedone 2, forming a Michael addition product. Subsequently, the nucleophilic attack of the 1,3-dicarbonyl compound on the activated Michael addition product takes place. This step is followed by an intramolecular cyclization process, leading to the formation of the benzoxanthenones compound, which corresponds to compound 3. In the absence of the W catalyst, the Michael addition products were found to be the major products formed in the reaction system. This suggests that the presence of the W catalyst plays a crucial role in shifting the reaction towards cyclization.

## Conclusions

The utilization of W/Cu@g-C_3_N_4_ as an environmentally friendly and efficient catalyst enabled the successful synthesis of benzoxanthenone and xanthene derivatives without the need for solvents, while the catalyst maintained its effectiveness upon reuse with good efficiency. The W/Cu@g-C_3_N_4_ nanocomposite was prepared under hydrothermal conditions by WCl_6_/CuCl_2_ supported on graphitic carbon nitride, which has several advantages, such as environmental safety of the catalyst and solvents, high catalytic efficiency, easy application, and high reaction speed. Using W/Cu@g-C_3_N_4_ as a catalyst also offers practical benefits, including its easy recyclability. After five times of use, no decrease in its catalytic performance was observed, indicating its high stability and cost-effectiveness for large-scale synthesis. Overall, the synthesis method using W/Cu@g-C_3_N_4_ provides an efficient, environmentally friendly, and practical approach for synthesizing benzoxanthenones and xanthenes derivatives (Supplementary Figures).

### Supplementary Information

Supplementary Figures.

## Data Availability

The data that support the findings of this study are available on request from the corresponding author.

## References

[1] Choudhary MI, Khan SN (2015). Xanthenes and their derivatives: Biological activities and recent advances in synthetic strategies. Life Sci..

[2] Zhang ZH, Wang HJ, Ren XQ, Zhang YY (2009). A facile and efficient method for synthesis of xanthone derivatives catalyzed by HBF4/SiO_2_ under solvent-free conditions. Chem. Monthly.

[3] Li P, Ma F, Wang P, Zhang Z (2013). Highly efficient low melting mixture catalyzed synthesis of 1,8-dioxo-dodecahydroxanthene derivatives. Chin. J. Chem..

[4] Azizi N, Abbasi F, Abdoli-Senejani M (2018). Natural acidic ionic liquid immobilized on magnetic silica: Preparation and catalytic performance in chemoselective synthesis of dicoumarols and substituted xanthene derivatives. ChemistrySelect.

[5] Liu X, Zhang H, Wang Y (2020). Recent advances in the synthesis and biological activities of xanthene derivatives. Eur. J. Med. Chem..

[6] Li JJ, Tao XY, Zhang ZH (2008). An effective bismuth trichloride-catalyzed synthesis of 1,8-dioxo-octahydroxanthenes phosphorus. Sulfur Silicon Relat. Elem..

[7] Xie F, Wang Y, Luo S (2019). Recent advances in synthetic strategies and biological activities of xanthenes and their derivatives. Curr. Org. Chem..

[8] Kumar A, Shaikh AA (2021). Xanthenes as a versatile scaffold in drug discovery: Recent advances. Eur. J. Med. Chem..

[9] Saeed A, Ashraf M (2019). Xanthenes: Synthetic strategies and medicinal applications. Mini-Rev. Med. Chem..

[10] Nandi GC, Samai S, Kumar R, Singh MS (2009). An efficient one-pot synthesis of tetrahydrobenzo[a]xanthene-11-one and diazabenzo[a]anthracene-9,11-dione derivatives under solvent free condition. Tetrahedron.

[11] Bansal P, Chaudhary GR, Kaur N (2015). An efficient and green synthesis of xanthene derivatives using CuS quantum dots as a heterogeneous and reusable catalyst under solvent free conditions. RSC Adv..

[12] Taheri A, Lai B, Yang J, Zhang J, Gu Y (2016). Facile synthesis of densely substituted chroman derivatives through Brønsted acid ionic liquid catalyzed three-component reactions of aromatic aldehydes, 1,1-diarylethylenes and nucleophiles. Tetrahedron.

[13] Chaudhary GR, Bansal P, Mehta NK (2014). Recyclable CuO nanoparticles as heterogeneous catalysts for the synthesis of xanthenes under solvent free conditions. RSC Adv..

[14] Azizi N, Shirdel F (2016). Task specific dicationic acidic ionic liquids catalyzed efficient and rapid synthesis of benzoxanthenones derivatives. J. Mol. Liquids.

[15] Das PJ, Das J (2015). Secondary amine based ionic liquid: An efficient catalyst for solvent free one pot synthesis of xanthenes and benzoxanthenes. RSC Adv..

[16] Pancholia S, Dhameliya TM, Shah P, Jadhavar PSJ, Sridevi P, Yogeshwari P, Sriram D, Chakraborti AK (2016). Benzo[d]thiazol-2-yl(piperazin-1-yl)methanones as new anti-mycobacterial chemotypes: Design, synthesis, biological evaluation and 3D-QSAR studies. Eur. J. Med. Chem..

[17] Zhang ZH, Zhang XN, Mo LP, Li YX, Ma FP (2012). Catalyst-free synthesis of quinazoline derivatives using low melting sugar–urea–salt mixture as a solvent. Green Chem..

[18] Rashidizadeh A, Ghafuri H, Azizi N (2020). Tandem oxidative amidation of alcohols catalyzed by copper modified well-ordered mesoporous graphitic carbon nitride. Solid State Sci..

[19] Chen X, Zhang J, Fu X, Antonietti M, Wang X (2009). Fe-g-C_3_N_4_-catalyzed oxidation of benzene to phenol using hydrogen peroxide and visible ligh. J. Am. Chem. Soc..

[20] Zhang J, Chen X, Takanabe K, Maeda K, Domen K, Epping JD, Fu X, Antonietti M, Wang X (2010). Synthesis of a carbon nitride structure for visible-light catalysis by copolymerization. Angew. Chem. Int. Ed..

[21] Shi L, Liang L, Wang F, Liu M, Chen K, Sun K, Zhang N, Sun J (2015). Higher yield urea-derived polymeric graphitic carbon nitride with mesoporous structure and superior visible-light-responsive activity. ACS Sustain. Chem. Eng..

[22] Zhang G, Zhang J, Wang X (2012). Polycondensation of thiourea into carbon nitride semiconductors as visible light photocatalysts. J. Mater. Chem..

[23] Soleymani Ahooie T, Azizi N, Yavari IM, Hashemi M (2018). Magnetically separable and recyclable g-C_3_N_4_ nanocomposite catalyzed one-pot synthesis of substituted imidazoles. J. Iran. Chem. Soc..

[24] Cha C, Shin SR, Annabi N, Dokmeci MR, Khademhosseini A (2013). Carbon-based nanomaterials: Multifunctional materials for biomedical engineering. ACS Nano.

[25] Zhang L, Ji L, Yao Z, Yan N, Sun Z, Yang X, Zhu X, Hu S, Chen L (2019). Facile synthesized Fe nanosheets as superior active catalyst for hydrogen storage in MgH2. Int. J. Hydrog. Energy.

[26] Yang CM, Chen TC, Verma D, Li LJ, Liu B, Chang WH, Lai CS (2020). Bidirectional all-optical synapses based on a 2D Bi_2_O_2_Se/graphene hybrid structure for multifunctional optoelectronics. Adv. Funct. Mater..

[27] Wang Y, Wang XC, Antonietti M (2012). Polymeric graphitic carbon nitride as a heterogeneous organocatalyst: From photochemistry to multipurpose catalysis to sustainable chemistry. Angew. Chem. Int. Ed..

[28] Zhang XL, Zheng C, Guo SS, Li J, Yang HH, Chen G (2014). Turn-on fluorescence sensor for intracellular imaging of glutathione using g-C3N4 nanosheet–MnO_2_ sandwich nanocomposite. Anal. Chem..

[29] Miller TS, Jorge AB, Suter TM, Sella A, Cora F, McMillan PF (2017). Carbon nitrides: Synthesis and characterization of a new class of functional materials. Phys. Chem. Chem. Phys..

[30] Zhang G, Zhang J, Zhang M, Wang X (2012). Polycondensation of thiourea into carbon nitride semiconductors as visible light photocatalysts. J. Mater. Chem..

[31] Huang D, Yan X, Yan M, Zeng G, Zhou C, Wan J, Cheng M, Xue W (2018). Graphitic carbon nitride-based heterojunction photoactive nanocomposites: Applications and mechanism insight. ACS Appl. Mater. Interfaces.

[32] Wang M-Q, Yang W-H, Wang H-H, Chen C, Zhou Z-Y, Sun S-G (2014). Pyrolyzed Fe–N–C composite as an efficient non-precious metal catalyst for oxygen reduction reaction in acidic medium. ACS Catal..

[33] Zhu M, Kim S, Mao L, Fujitsuka M, Zhang J, Wang X, Majima T (2017). Metal-free photocatalyst for H2 evolution in visible to near-infrared region: Black phosphorus/graphitic carbon nitride. J. Am. Chem. Soc..

[34] Di JQ, Zhang M, Chen YX, Wang JX, Geng SS, Tang JQ, Zhang ZH (2021). Copper anchored on phosphorus g-C3N4 as a highly efficient photocatalyst for the synthesis of N-arylpyridin-2-amines. Green Chem..

[35] Wang X, Maeda K, Thomas A, Takanabe K, Xin G, Carlsson JM, Domen K (2009). A metal-free polymeric photocatalyst for hydrogen production from water under visible light. Nat. Mater..

[36] Xu Y, Wang X, Antonietti M, Li H (2010). Highly efficient photocatalytic hydrogen production from water using visible-light-sensitive graphene-like g-C3N4. J. Am. Chem. Soc..

[37] Zhang G, Zhang Y, Zhang B, Ma L, Fang J (2015). Graphitic carbon nitride: Synthesis, properties, and applications in catalysis and photocatalysis. Chem. Commun..

[38] Dong F, Zhao Z, Sun Y, Wang X (2015). Graphitic carbon nitride based nanocomposites: A review. Nanoscale.

[39] Wang Y, Wang X, Antonietti M, Yu SH (2012). A metal-free polymeric photocatalyst for hydrogen production from water under visible light. J. Mater. Chem..

[40] Yang H, Xu L, Li Y, Liu H, Wu X, Zhou PN, Graham JD, Yu W (2023). FexO/FeNC modified activated carbon packing media for biological slow filtration to enhance the removal of dissolved organic matter in reused water. J. Hazard. Mater..

[41] Huang X, Song J, Hua M, Chen B, Xie Z, Liu H, Zhang Z, Meng Q, Han B (2021). Robust selenium-doped carbon nitride nanotubes for selective electrocatalytic oxidation of furan compounds to maleic acid. Chem. Sci..

[42] Zhao R, Wang Y, Ji G, Zhong J, Zhang F, Chen M, Tong S, Wang P, Wu Z (2023). Partially nitrided ni nanoclusters achieve energy-efficient electrocatalytic CO_2_ reduction to CO at ultralow overpotential. Adv. Mater..

[43] Azizi N, Khajeh M, Alipour M (2014). Rapid and selective oxidation of alcohols in deep eutectic solvent. Ind. Eng. Chem. Res..

[44] Azizi N, Saidi MR (2003). Lithium perchlorate diethyl ether solution: A highly efficient media for the abramov reaction. Phosphorus Sulf. Silicon Relat. Elem..

[45] Azizi N, Yadollahy Z, Rahimzadeh-Oskooee A (2014). An atom-economic and odorless thia-Michael addition in a deep eutectic solvent. Tetrahedron Lett..

[46] Venu Madhav Y, Thirupathi Reddy P, Reddy N (2009). Cellulose sulfuric acid: An efficient biodegradable and recyclable solid acid catalyst for the one-pot synthesis of aryl-14H-dibenzo[a.j]xanthenes under solvent-free conditions. J. Mol. Catal. A Chem..

[47] Madhav JV, Kuarm BS, Rajitha B (2008). Dipyridine cobalt chloride: A novel and efficient catalyst for the synthesis of 14-aryl 14H-dibenzo[a, j]xanthenes under solvent-free conditions”. Arkivoc.

[48] Amini MM, Seyyedhamzeh M, Bazgir A (2007). Heteropolyacid: An efficient and eco-friendly catalyst for the synthesis of 14-aryl-14H-dibenzo[a, j]xanthene. Appl. Catal. A Gen..

[49] Patil MS, Palav AV, Khatri CK, Chaturbhuj GU (2017). Rapid, efficient and solvent-free synthesis of (un)symmetrical xanthenes catalyzed by recyclable sulfated polyborate. Tetrahedron Lett..

[50] Niknam K, Panahi F, Saberi D, Mohagheghnejad M (2010). Silica-bonded S-sulfonic acid as recyclable catalyst for the synthesis of 1,8-dioxo-decahydroacridines and 1,8-dioxo-octahydroxanthenes. J. Heterocycl. Chem..

[51] Harichandran AS, David S (2014). Synthesis and characterization of phosphate anchored MnO_2_ catalyzed solvent free synthesis of xanthene laser dye. J. Mol. Catal. A Chem..

[52] Amoozadeh A, Rahmani S, Bitaraf M, Abadi FB, Tabrizian E (2016). Nano-zirconia as an excellent nano support for immobilization of sulfonic acid: A new, efficient and highly recyclable heterogeneous solid acid nanocatalyst for multicomponent reactions. New J. Chem..

[53] Jing-Jun W, Chun W, Qiu-Hua T, Ran-Xiao L, Hai-Yan L, Qing M (2008). An efficient green synthesis of xanthenedione derivatives promoted by acidic ionic liquid. Heteroat. Chem..

[54] Fang D, Yang JM, Liu Z-L (2011). (2011) Eco-friendly synthesis of 1,8-dioxo-octahydroxanthenes catalyzed by ionic liquid in aqueous media. J. Heterocycl. Chem..

[55] Zhang ZH, Tao XY (2008). 2,4,6-Trichloro-1,3,5-triazine-promoted synthesis of 1,8-dioxo- octahydroxanthenes under solvent-free conditions. Aust. J. Chem..

[56] Pore S, Patil DD (2010). Envirocat EPZ-10: A solid acid catalyst for the synthesis of 1,8-dioxo-octahydroxanthenes in aqueous medium. Synth. Commun..

[57] Karhale S (2020). Grafting of sulphamic acid on functionalized sawdust: A novel solid acid catalyst for the synthesis of 1,8-dioxo-octahydroxanthenes. Res. Chem. Intermed..

[58] Aher DS, Khillare KR, Shankarwar SG (2021). Incorporation of Keggin-based H3PW7Mo5O40into bentonite: Synthesis, characterization and catalytic applications. RSC Adv..

[59] Maheswari CS, Ramesh RL (2017). A. One-pot synthesis of symmetrical and unsymmetrical acridine sulfonamide derivatives catalyzed by p-TSA. Res. Chem. Intermed..

