# Supplementary material for: Streamlining efficient and selective synthesis of benzoxanthenones and xanthenes with dual catalysts on a single support
Source: Sci Rep. 2023 Sep 30;13:16469. doi: 10.1038/s41598-023-43746-y (PMC10542355; doi:10.1038/s41598-023-43746-y)
Supplement: Supplementary file 1 — Supplementary Figures. [file 41598_2023_43746_MOESM1_ESM.docx]

**supporting material**

Streamlining Efficient and Selective Synthesis of Benzoxanthenones and Xanthenes with Dual Catalysts on a Single Support

Najmedin Azizi^a*^ Fezzeh Farzaneh, Elham Farhadi

^a^ Chemistry and Chemical Engineering Research Center of Iran, P.O. Box 14335-186, Tehran, Iran

E-mail: [azizi@ccerci.ac.ir](mailto:azizi@ccerci.ac.ir)

**
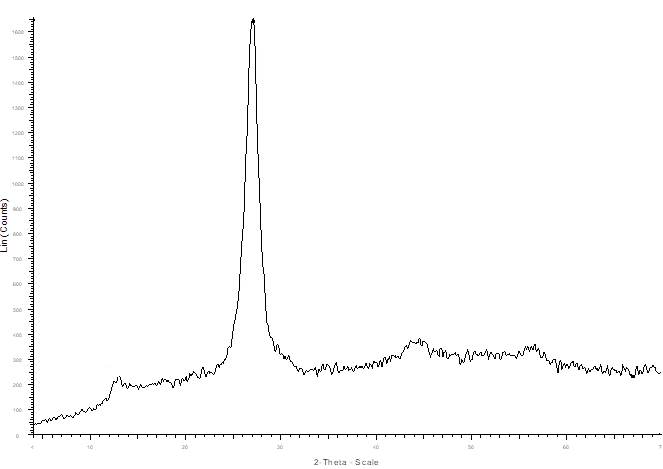
**

Figure S1 XRD patterns of the g-C_3_N_4_


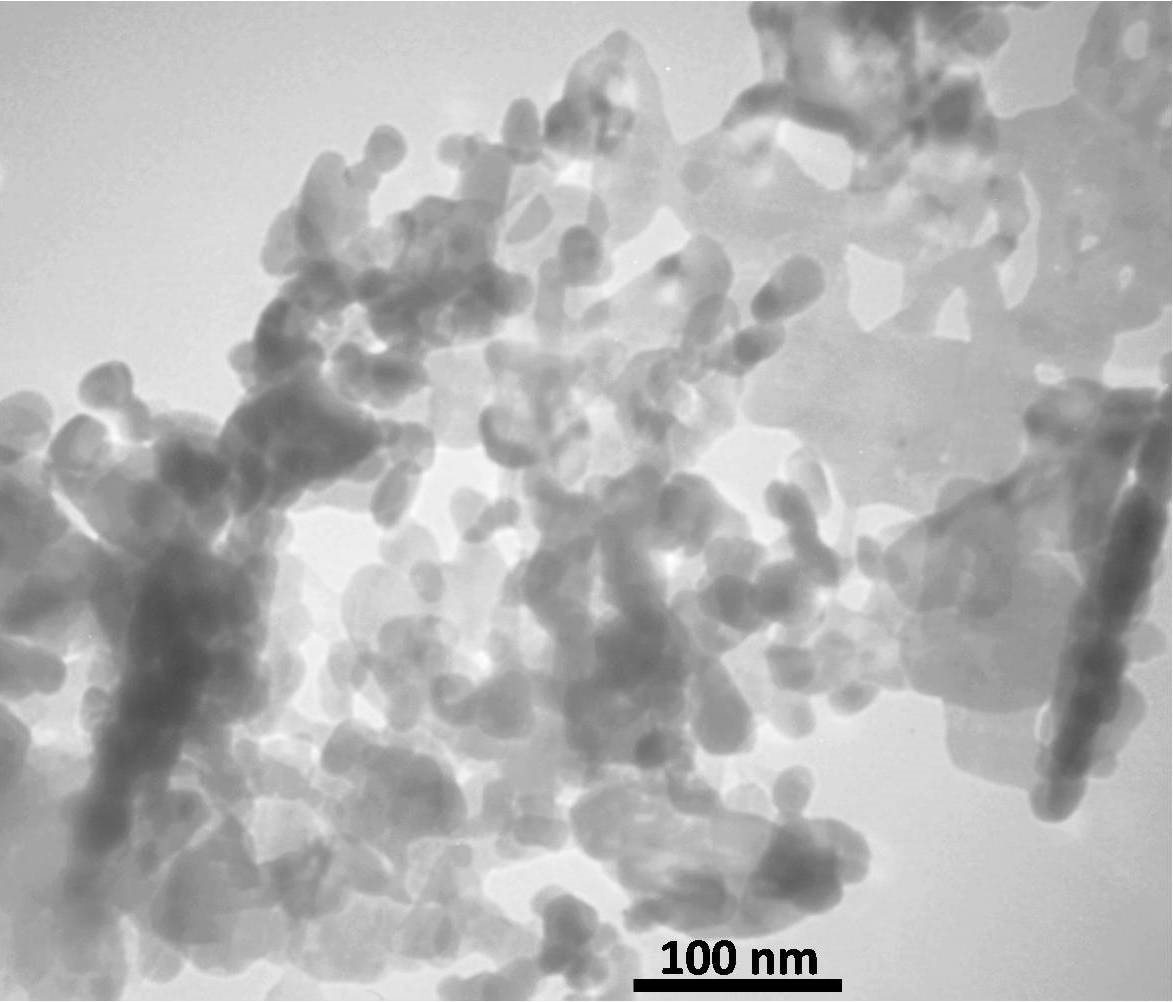


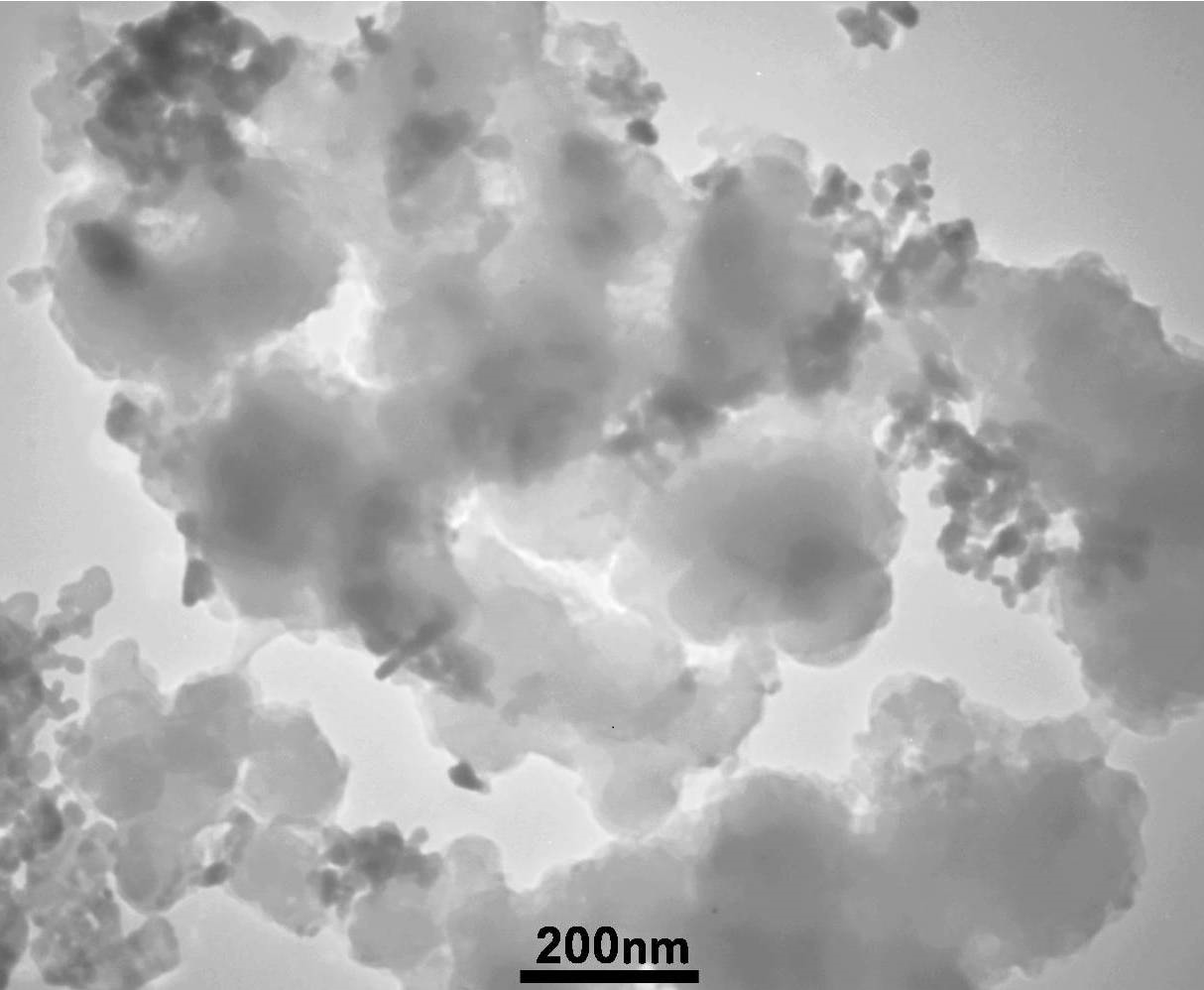


Figure S2 The TEM image of five cycled reused W/Cu@g-C_3_N_4_


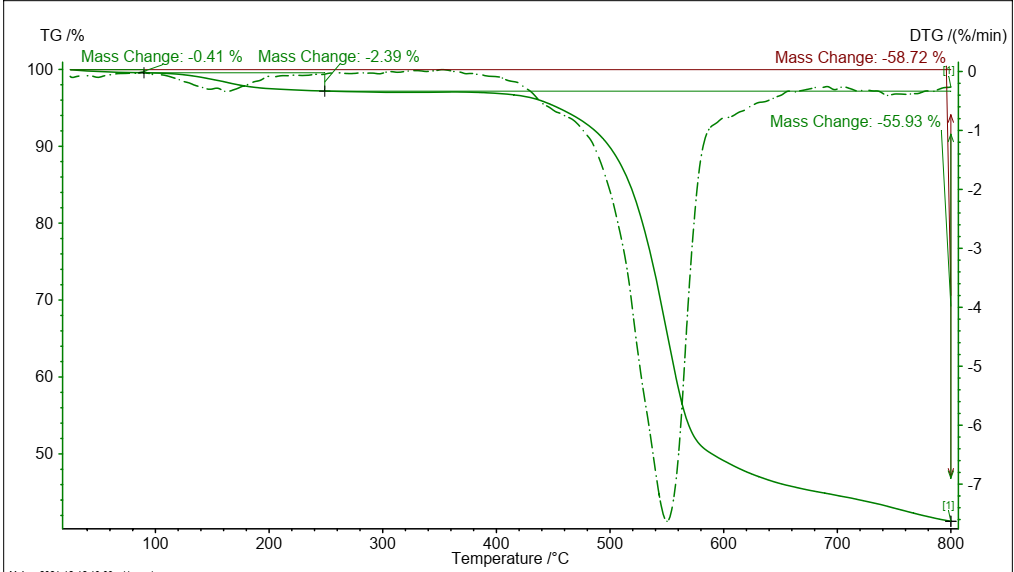


**Figure S3**  TGA diagram of the five cycled reused W/Cu@g-C3N4


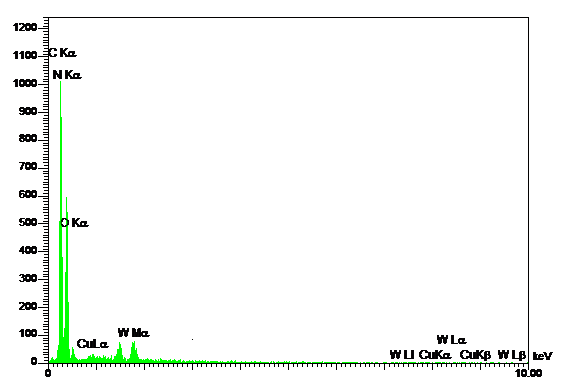


Figure S4 EDX analysis five cycled reused W/Cu@g-C3N4


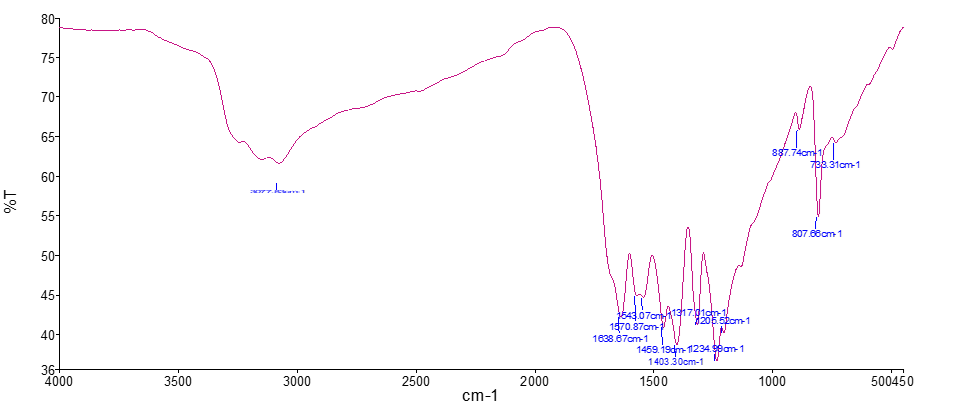


Figure S5 FTIR analysis five cycled reused W/Cu@g-C3N4


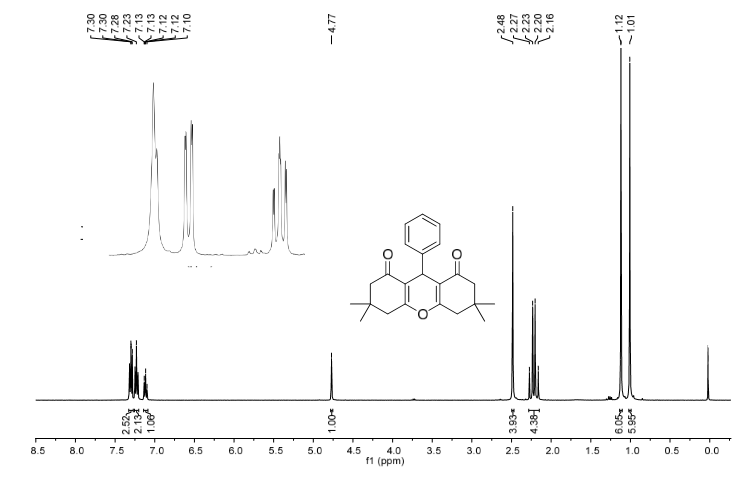


Figure S6 ^1^H NMR spectra of compound **3a**


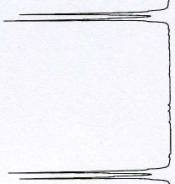

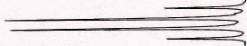

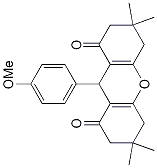

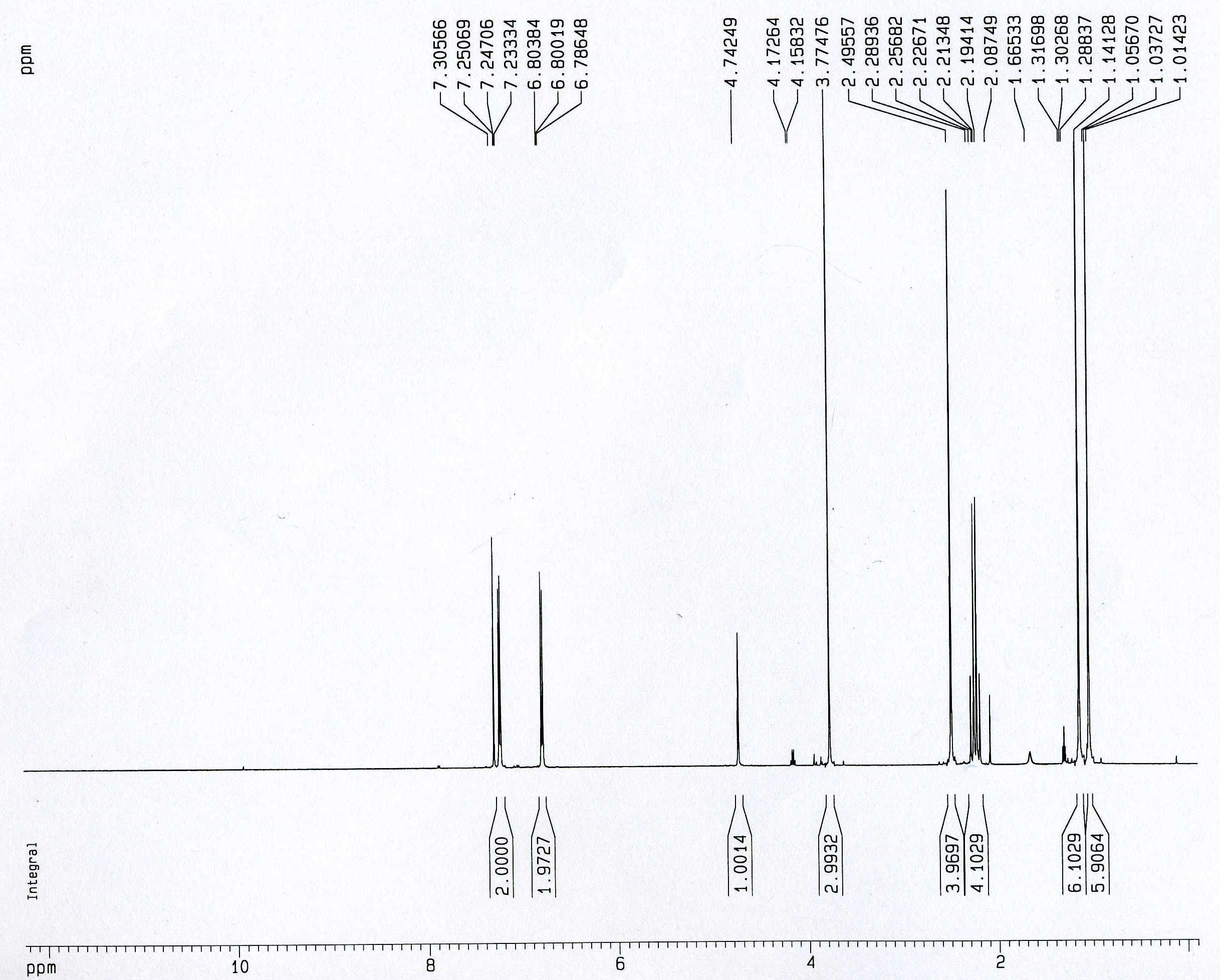


Figure S7 ^1^H NMR spectra of compound **3e**


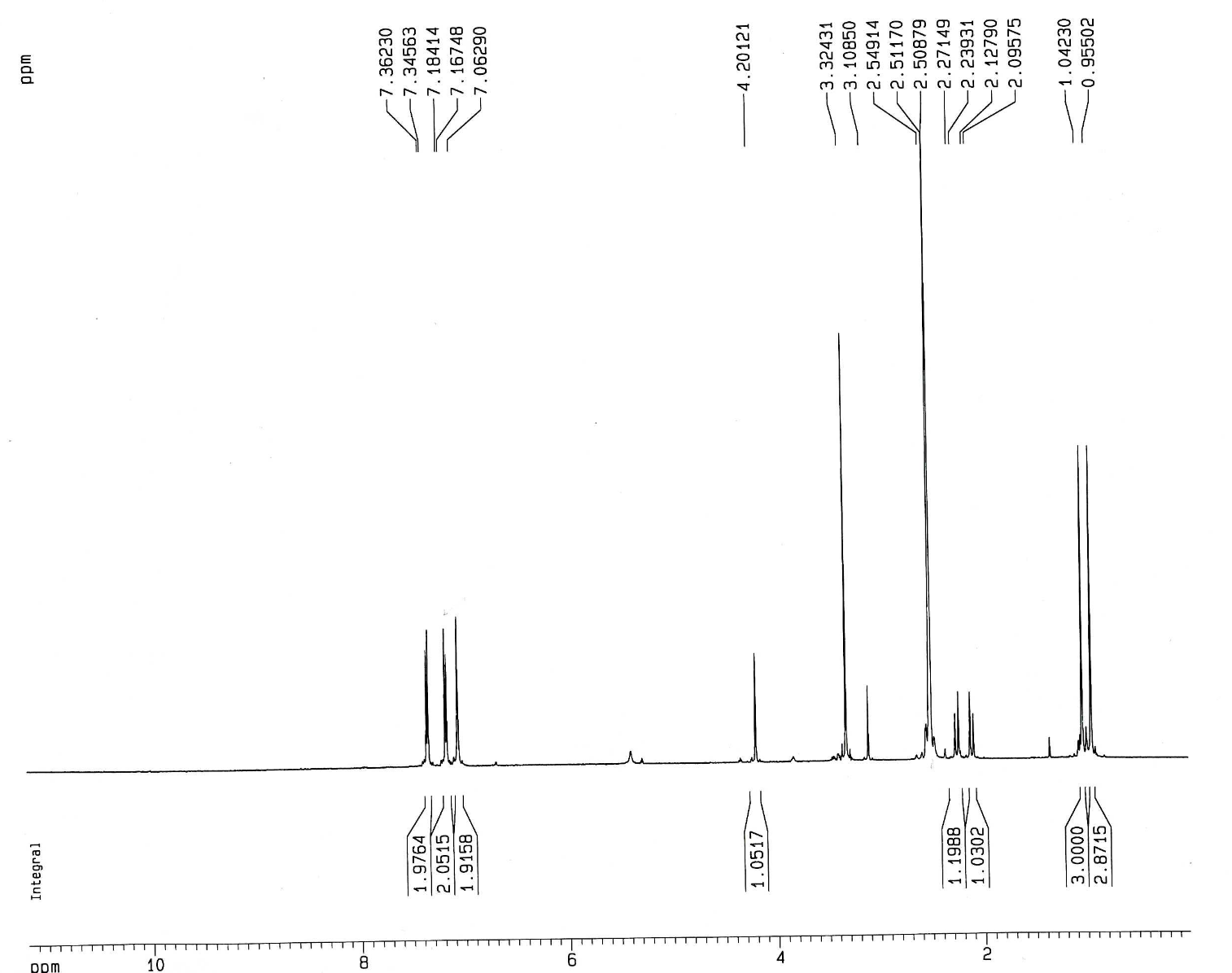

Figure S8 ^1^H NMR spectra of compound **3b**


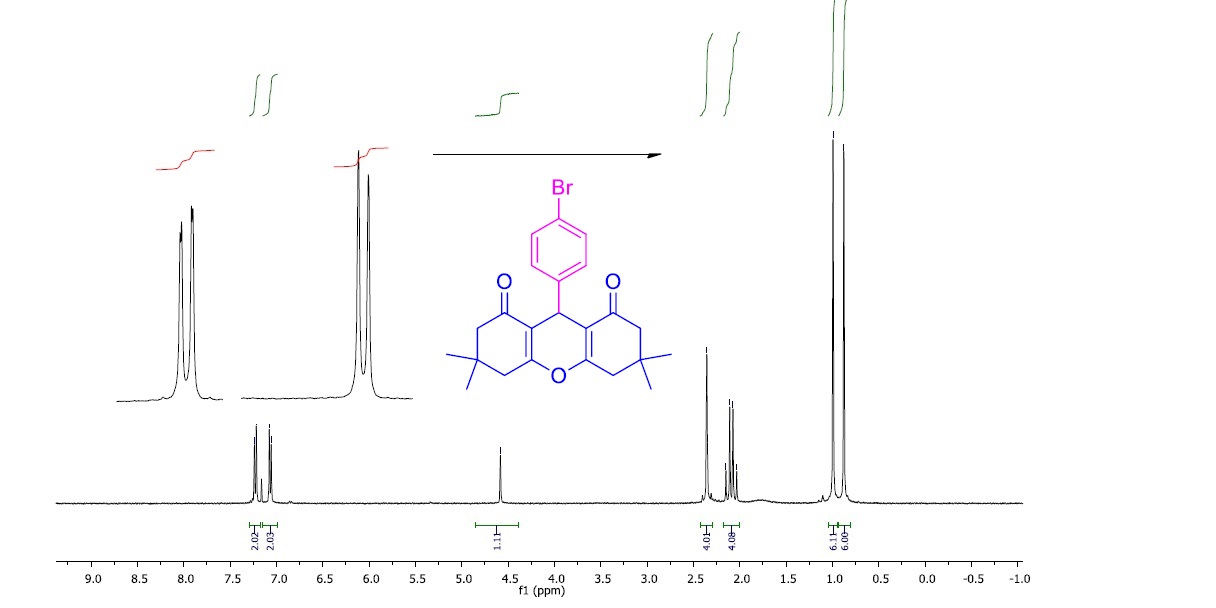


Figure S9 ^1^H NMR spectra of compound **3c**


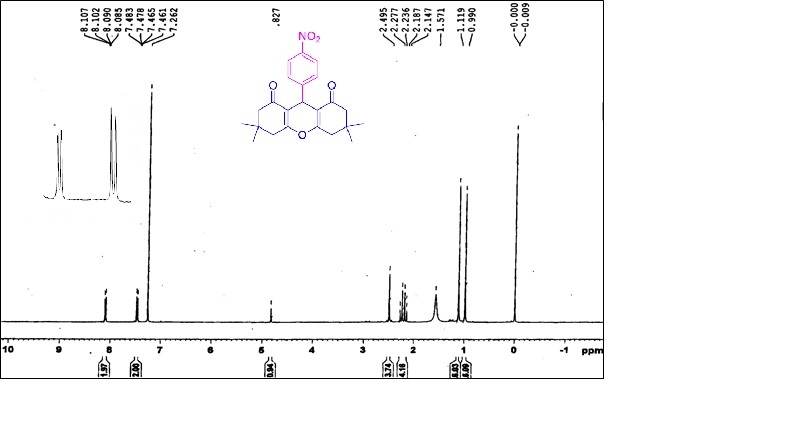


Figure S10 ^1^H NMR spectra of compound **3f**


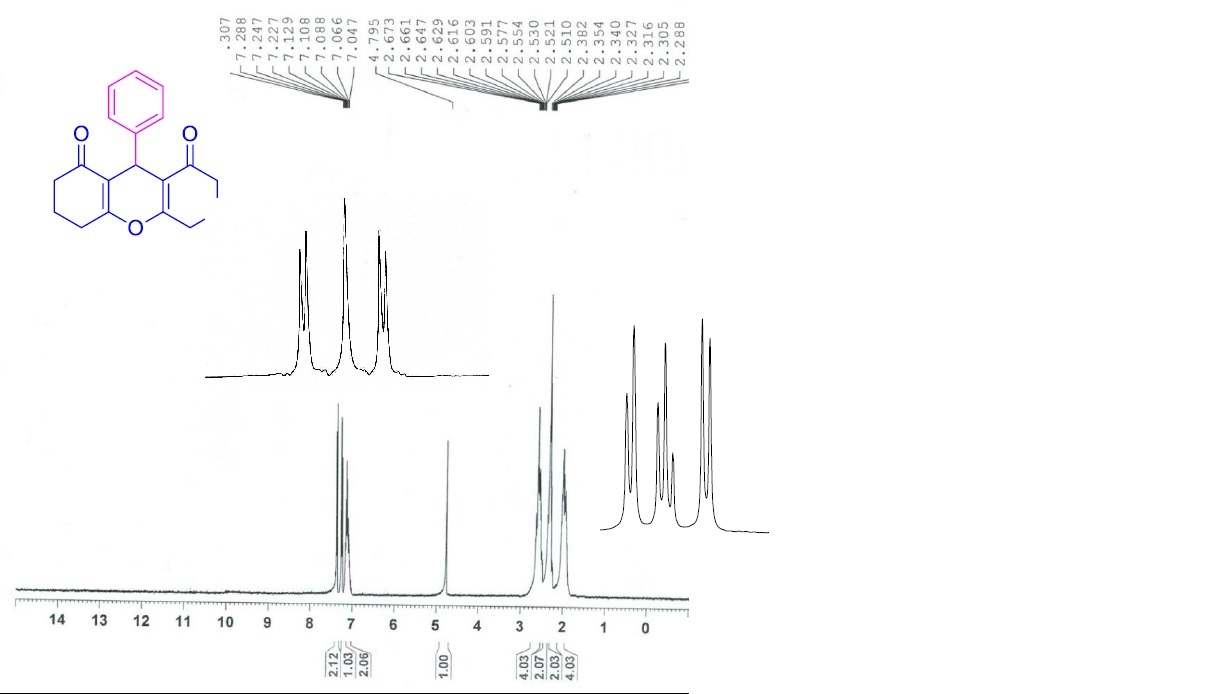


Figure S11 ^1^H NMR spectra of compound **3h**


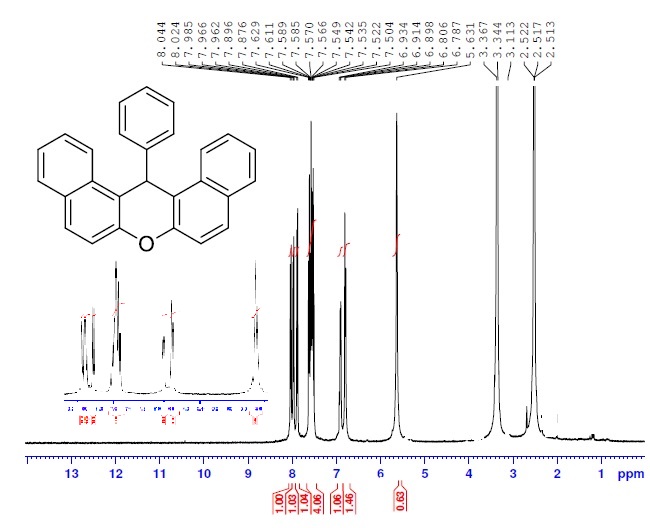


Figure S12 ^1^H NMR spectra of compound **5a**


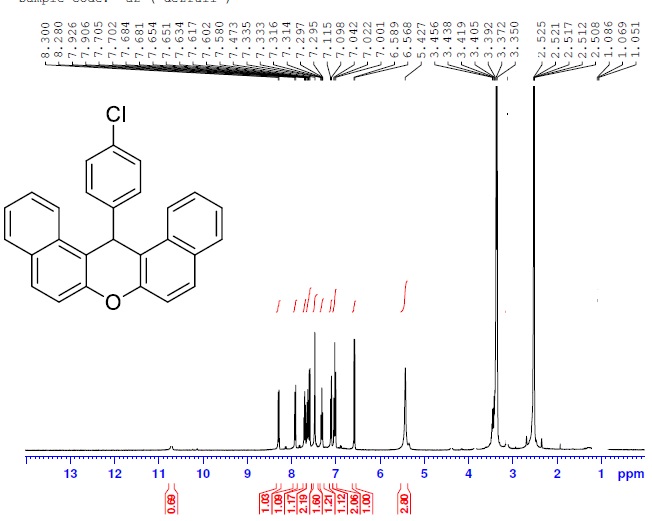


Figure S13 ^1^H NMR spectra of compound **5e**
